# Using qualitative interviews to identify patient-reported clinical trial endpoints and analyses that are the most meaningful to patients with advanced breast cancer

**DOI:** 10.1371/journal.pone.0280259

**Published:** 2023-01-17

**Authors:** Emuella Flood, Anna Krasnow, Cecilia Orbegoso, Stella Karantzoulis, Julie Bailey, Solène Bayet, Arthur Elghouayel, Andrew Foxley, Roberto Sommavilla, Gaia Schiavon

**Affiliations:** 1 AstraZeneca plc, Patient-Centered Science, Gaithersburg, Maryland, United States of America; 2 IQVIA Real World Solutions, Patient-Centered Solutions, London, United Kingdom; 3 AstraZeneca plc, R&D Oncology, Cambridge, United Kingdom; 4 IQVIA Real World Solutions, Patient-Centered Solutions, New York, New York, United States of America; 5 IQVIA Real World Solutions, Patient-Centered Solutions, Courbevoie, France; Örebro University Faculty of Medicine and Health: Orebro universitet Fakulteten for medicin och halsa, SWEDEN

## Abstract

**Background:**

Designing clinical trials with the emphasis on the patient-centered approach and focusing on clinical outcomes that are meaningful to patients is viewed as a priority by drug developers, regulatory agencies, payers, clinicians, and patients. This study aimed to capture information on clinical trial endpoints that would be most important and relevant for patients with advanced breast cancer, based on patient-reported outcomes.

**Methods:**

Patients with either advanced triple-negative breast cancer [TNBC] and a maximum of two lines of systemic therapy or hormone receptor-positive/human epidermal growth factor receptor 2-negative [HR+/HER2−] breast cancer and a maximum of three lines of systemic therapy, participated in semi-structured concept elicitation interviews. Concept saturation was assessed. A sign, symptom, or impact was defined as “salient” if mentioned by ≥ 60% of participants, with an average bother rating of ≥ 5 (0–10 Scale). Participants were also asked about treatment priorities and to evaluate hypothetical scenarios showing different health-related functioning and quality-of-life treatment outcomes, using graphical representations.

**Results:**

Thirty-two participants (97% women; aged 29+ years) with TNBC (n = 17) or HR+/HER2− breast cancer (n = 15) provided generally similar reports on symptom experience, with fatigue and pain being most salient, though importance of certain treatment-related symptoms varied between the two groups. Patients reported consistent perspectives on the importance of treatment outcomes: when considering a new treatment, they prioritized efficacy of the therapy, acceptable tolerability, stability, predictability of symptoms over time, and the duration of preserved health-related quality of life and physical functioning. The meaningful difference in preserved physical functioning was 2–3 months for 46% of participants with TNBC, whereas for most participants with HR+/HER2− breast cancer it started from 6–7 months. Both groups of participants found it easier to accept some toxicity at the beginning of therapy if it was followed by improvement, as opposed to improvement followed by deterioration.

**Conclusion:**

The results may help to inform the design of patient-centered clinical trials, to interpret health-related quality of life and/or patient-reported outcomes, and to optimize care for patients with advanced breast cancer.

## Introduction

Designing clinical trials with a focus on a core set of clinical outcomes that are meaningful to patients is viewed as a priority by researchers, regulators, payers, and policymakers [1, 2]. The current period of rapid progress in drug development has led to the introduction of novel therapies and combinations of multiple drug classes that are characterized by diverse toxicities and adverse events. During this time, identification of appropriate patient-centered endpoints and their accurate characterization has become increasingly important in assessing the risks and benefits of new treatments [3]. The 21st Century Cures Act and the US Food and Drug Administration (FDA) guidelines emphasize the importance of direct incorporation of patients’ perspectives into the drug development process [4]. In 2014, the American Society of Clinical Oncology (ASCO) Cancer Research Committee underscored the need to measure health-related quality of life (HRQoL) in clinical trials; at the same time, the Committee recognized that HRQoL is difficult to measure and to interpret, noting particularly the challenge in defining clinically informative measures [5]. Since the preferences of patients for various endpoints are not sufficiently understood and patients’ knowledge about treatment options may be limited, the implementation of a patient-centered approach in clinical studies might benefit from a systematic approach to receiving and analyzing feedback from patients [6, 7].

In this study, we focus on breast cancer − a disease accounting in 2020 for 11.7% of all newly diagnosed cancers worldwide and the leading cause of cancer death among women [8, 9]. Specifically, we focus on two subgroups of patients with advanced (unresectable locally advanced or metastatic) breast cancer: triple-negative breast cancer (TNBC) and hormone receptor-positive/human epidermal growth factor receptor 2-negative (HR+/HER2−) breast cancer. These populations were chosen because they represent two distinct types of this complex heterogeneous disease: they have dissimilar prognoses, are treated according to different standards of care, and are typically enrolled in different clinical trials. TNBC is an aggressive form of breast cancer, accounting for 15−20% of cases worldwide [10, 11]. It is characterized by an earlier age of onset, greater metastatic potential, and poorer clinical outcomes than HR+ or HER2+ breast cancer [12, 13]; TNBC is treated primarily with chemotherapy, which is associated with higher toxicity and reduced HRQoL than endocrine therapy [14].

HR+/HER2−breast cancer, representing around 70% of cases, is usually diagnosed during the early stage of the disease and typically treated with curative intent, centered around surgery and adjuvant endocrine therapy, which are generally well tolerated [15]. At the metastatic stage, HR+/HER2− breast cancer is treated with serial endocrine therapy-based regimens, in combination with targeted therapy, until the disease becomes endocrine-resistant [16], followed by transition to single-agent chemotherapy [15].

Published qualitative interview studies have documented the signs, symptoms, and impacts of patients experiencing advanced or metastatic breast cancer [17–19]. Whilst patients commonly experience fatigue/tiredness, hair loss, general pain, lump in breast, and nausea, there is often high variability in other signs, symptoms, and impacts, due to the various sites of metastasis, differences in treatments, and the patient’s disease history [17–21]. In addition, there is a lack of data on the lived experience of patients with TNBC.

The information needs of patients with breast cancer have also been documented, identifying differences in needs according to place in the cancer journey (e.g pre-diagnosis, diagnosis and treatment, or survivorship) and disease stage (e.g. advanced breast cancer), as well as age and other demographic characteristics [22]. Information needs reported in advanced breast cancer include symptom and side-effect management, as well as information on treatment options (including clinical trials) and prognosis [23, 24]. However, a specific understanding of patient experiences and information needs for treatment decision-making in advanced TNBC and HR+/HER2– breast cancer is desirable. Interpretation of differences and similarities could allow researchers to draw conclusions more confidently about generalizability [20, 22].

The relatively few studies focusing on patient preference assessments for advanced breast cancer report that patients value acceptable levels of HRQoL, and in scenarios of advanced and metastatic disease, patients are willing to face the risks of side effects if survival is extended [1, 25, 26]. For patients with TNBC and HR+/HER2– breast cancer, a better understanding of their preferences, especially regarding HRQoL when considering perceived treatment-related side effects, may result in improved healthcare decision-making and development of oncological therapies for advanced and metastatic cancer. Identifying patient’s perceptions of which response to treatment is more favorable and why, as well as understanding which representations of clinical trial data are most meaningful to support treatment decision-making, can result in more patient-centric development of oncological therapies that improve patient HRQoL.

Here, we describe a study aiming to obtain a comprehensive overview of the patient experience of advanced TNBC and HR+/HER2− breast-cancer-related signs, symptoms, and impacts, and to utilize hypothetical scenarios to examine what information is most important for patients with advanced breast cancer when they think about starting a new treatment, and to inform what clinical trial endpoints are most relevant and meaningful for them. Hearing from patients directly about their experiences enhances our understanding of how breast cancer manifests in daily lives [17]. Gathering and analyzing inputs about meaningful outcomes from two important populations of patients with breast cancer who have distinct disease- and treatment-related experiences may inform clinical trials not only in breast cancer, but also in other areas of oncology where it may not be appropriate to assume that organ class rather than other factors is the primary determinant of how patients will consider the impact of a new therapy.

Qualitative research methods were utilized to collect in-depth information pertaining to patient experiences of living with TNBC or HR+/HER–breast-cancer as well as in understanding their treatment priorities; these methods provide rich insight and context on the nuances and heterogeneity of the lived experience of the disease and treatment and drivers behind treatment priorities that may not be described quantitatively [27, 28]. Additionally quantitative data collection was also integrated to complement the qualitative data collection and its analysis; these data supported the generalizability of the results, assuaged concerns of researcher bias, and provided alternative perspectives to interpreting the results [29]. This mixed methods approach was taken with this study in order to more fully capture the patient experience of living with TNBC or HR+/HER–breast-cancer, and to discover the themes surrounding patients’ reasoning behind their preferences when deciding on a new treatment. This approach also allowed for a more in-depth explanation of how patients experience their most bothersome or interfering signs, symptoms, and impacts, and how meaningful they perceive certain HRQoL outcomes and clinical trial endpoints to be when making a treatment decision.

## Methods

This was a qualitative patient interview study, which captured information on clinical trial endpoints relevant to patients with advanced breast cancer. The 32-item Consolidated Criteria for Reporting Qualitative Research (COREQ) checklist was followed in the reporting of the study [30].

### Study design

The study’s primary objective was to understand the patient experience of living with TNBC or HR+/HER2− breast cancer. The principle of concept saturation of *a priori* and newly emerged concepts in each group (TNBC and HR+/HER2– breast-cancer patients) was used to assess the adequacy of the sample size based on the primary objective [31]. A sample size of 12–25 participants is typically adequate for concept elicitation interviews and for reaching saturation of concepts [32, 33]. The study’s secondary objectives were 1) to determine how patients make decisions to start a new treatment and 2) to understand which clinical trial endpoints are most relevant and meaningful to patients and why. This study was conducted with a mixed methods approach, in order to provide an understanding of which signs, symptoms, and impacts are experienced by patients as well as their responses around hypothetical scenarios and clinical trial endpoints, while also providing more context to the data collected with an in depth understanding of patient experiences and preferences through patient quotes. An initial literature search and qualitative interviews with clinicians were performed to guide the patient interviews. The study was approved by the New England Independent Review Board in the USA (IRB#: 120190258) and by the Health Research Authority in the UK (REC Reference: 19/LO/1653 & IRAS project ID: 271972). Informed consent was obtained in writing during the International Classification of Functioning, Disability and Health (ICF) process and confirmed again orally at the beginning of each interview from all individual participants included in the study.

### Participants

A cross-sectional design was used to collect qualitative data from participants in the UK and USA with a confirmed diagnosis (as determined by mammogram, positron emission tomography, and core needle biopsy) of advanced TNBC or HR+/HER2− breast cancer using a convenience sampling strategy. A maximum of two lines of previous systemic therapy was allowed for participants with TNBC and of three lines for participants with HR+/HER2− breast cancer. Participants were recruited by email, telephone, or in person via a patient advocacy group (Living Beyond Breast Cancer) and medical marketing agency (Global Perspectives) in the USA or through collaboration with physicians at the Royal Cornwall Hospitals NHS Trust for patients in the UK. Participants were then sent a link to an online screening form, via a patient advocacy group representative or site personnel. Participants were compensated USD 150 in the USA and GBP 125 in the UK.

### Data collection

A targeted literature review and semi-structured interviews with six expert clinicians specialized in treating TNBC (n = 3) or HR+/HER2− breast cancer (n = 3) provided foundational information to develop a semi-structured patient interview guide, including hypothetical scenarios showing HRQoL outcomes during a trial, specifically for this study. Participating clinicians were selected based on their expertise and were approached to participate in an interview by the research team. Patient sociodemographic and clinical data were collected by the advocacy group or treating physician before commencement of patient interviews (via the confirmation of diagnosis form) and via the interview online screening survey. All patients meeting eligibility criteria were invited to participate in this study. Those not meeting eligibility criteria were thanked for their time and were not invited to participate. After providing electronic informed consent, participants were interviewed via telephone by trained interviewers; interviews were accompanied by an online screen share for visual content and each interview lasted 60–90 minutes.

The telephone interviews with participants were conducted by five interviewers (four female and one male; authors SK, JB, AK, SB, and AE; educated to BA, MSc, or PhD level; all were trained and experienced in conducting patient concept elicitation interviews; other project team members were occasionally present on the calls for training purposes [with permission from the patient]). There was no relationship between interviewers and participants before study commencement Participants were made aware that the output of the interview might contribute to our understanding of patient experiences of breast cancer and its treatment, and what treatment outcomes are most meaningful, which could contribute to the design of future research studies.

Data collection and analysis were conducted through an iterative process in several waves (each wave included 2–3 patients). To evaluate concept saturation, concepts derived from each wave, both *a priori* and newly emerged during interviews, were compared with concepts from previous waves to determine if any new concepts were identified from interviews. If new concepts from the next wave appeared, saturation had not yet been achieved. Regular team meetings were held to review and to discuss participant feedback, and the guide was refined over the course of the interviews to add exploration of new concepts elicited during each wave.

The participant interview content was divided into three sections. Section 1 was devoted to concept elicitation, in which participants were first given the opportunity to mention signs, symptoms, and impacts related to TNBC or HR+/HER2− breast cancer spontaneously. The moderator then used a list of concepts from the previously conducted literature review to probe on additional concepts. New concepts were identified during the discussion and explored in interviews with subsequent participants. Bother ratings were collected for each concept mentioned by the participant both spontaneously and after probing. A sign, symptom, or impact was defined as “salient” if it was mentioned by more than 60% of participants and with an average bother rating of at least five on the bothersome scale (0 = not bothersome; 10 = extremely bothersome).

Section 2 focused on treatment priorities and involved two different exercises using hypothetical scenarios to explore patient-tailored treatments and treatment outcomes in the course of a trial. During the patient-tailored treatment exercise, participants were asked to imagine a new therapy personalized to their needs and to explain how it would make them feel. Probes were included to elicit the symptoms that participants hoped would be improved and why, what side effects they hoped to avoid, and what questions they would ask their physician to help them to make an informed decision about a new treatment.

**Fig 1:**
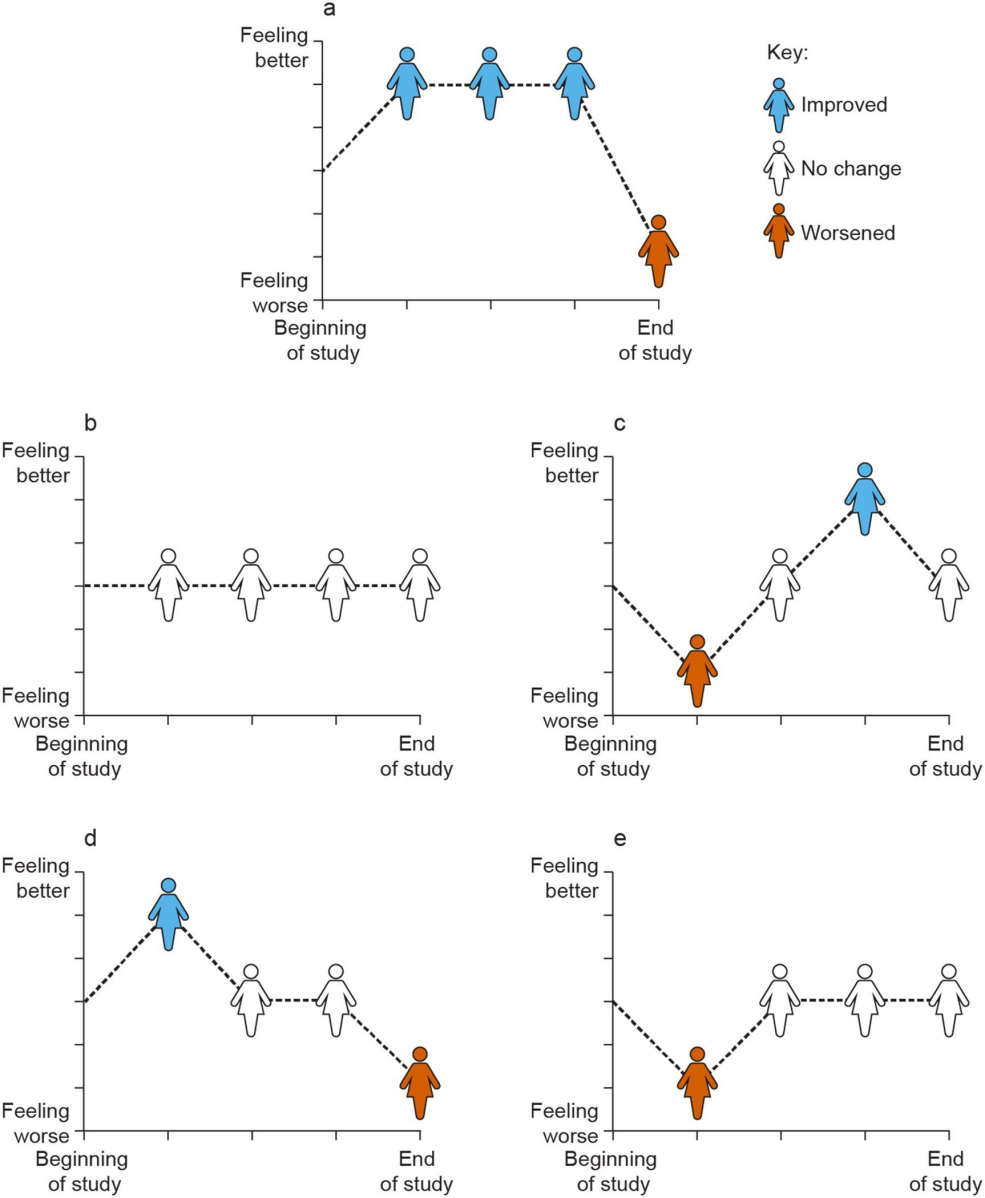
Trial responses for HRQoL. Graphical presentations of hypothetical scenarios showing HRQoL outcomes during a trial: **(a)** a period of improvement followed by worsening at the end of the trial; **(b)** no change in HRQoL over the course of the trial; **(c)** initial worsening followed by improvement and return to baseline; **(d)** initial improvement, a period of stability on the baseline level, followed by worsening; **(e)** initial worsening followed by a sustained period of stability at the baseline level.

During the treatment outcomes exercise, participants were asked to choose between five different predefined hypothetical scenarios representing potential HRQoL outcomes during a trial, presented in graphical format (Fig 1). The variants of trial outcomes were always presented in pairs, and all participants viewed the same four pairs of outcomes in the same order. Participants were asked to describe in their own words which of the two scenarios in each pair represented a better experience during a trial and to explain their decision. Probes were focused on factors that drove patients to choose their preferred scenario. The use of qualitative methods (e.g., interviews) to capture patient preferences is documented in the IMI PREFER recommendations [34].

**Fig 2:**
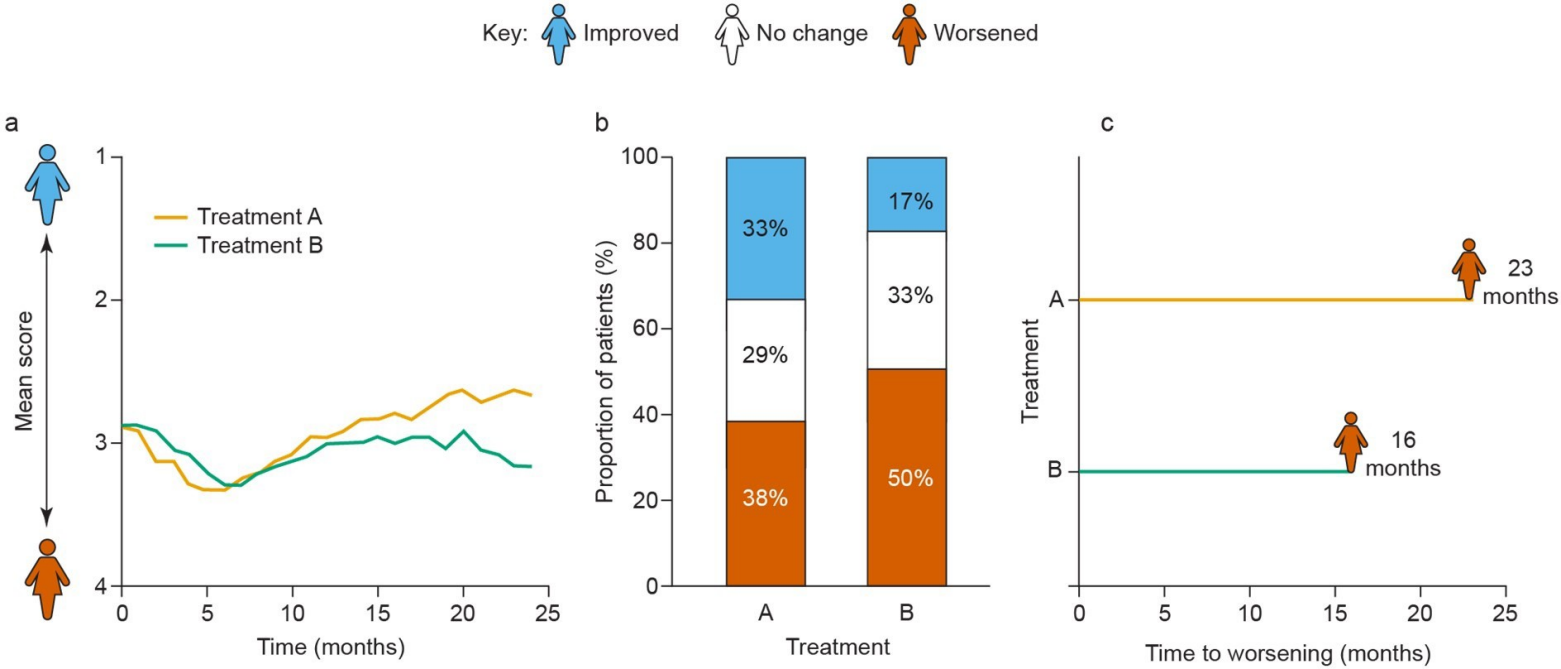
Endpoint preferences: Hypothetical trial data on physical functioning. The three endpoint scenarios depicting outcomes for two different groups of patients treated with hypothetical drugs A and B: **(a)** time-dependence of a mean score for physical function; **(b)** percentage of patients experiencing improvement, no change, or worsening in physical functioning over the course of treatment; (**c)** median time to worsening of physical functioning.

Section 3 focused on an endpoint preference and involved three hypothetical scenarios depicting a change in physical function (ability to do strenuous activities) during a trial for two groups of patients treated with drugs A and B: 1) a change in mean score for physical function in the course of treatment (Fig 2A); 2) percentage of patients experiencing improvement, no change, or worsening in physical functioning during a trial (Fig 2B); and 3) median time to deterioration (TTD), formulated in patient-friendly language as “time to worsening” of physical functioning (Fig 2C). “Time to worsening” was described to participants as the number of months that it took for half of the trial participants to experience a worsening in their ability to perform strenuous activities. A worsening was defined as an increase of 1 point or more on the physical function score since the beginning of the trial, on a scale of 1–4 (“Do you have any trouble doing strenuous activities, like carrying a heavy shopping bag or a suitcase?”: 1 = not at all; 2 = a little; 3 = quite a bit; 4 = very much). Each participant explained, in their own words, their understanding of the data presented in each graph and how they would use this information to make decisions regarding their own treatment; they also provided an overall rating on a scale of 0–10 (0 = not at all meaningful; 10 = very meaningful) for how personally significant the information in each graph would be for making a decision. Participants were also asked to describe the minimal difference in “time to worsening” between treatments A and B that would be consequential to them. No further interviews were conducted, and participants did not provide feedback on the findings.

### Analyses

Out of the 312 patients with TNBC and HR+/HER2− breast cancer who actively decided to participate in screening (via clicking the link), 212 (68%) initially joined based on self-report and consented to participation, 180 (58%) of those 212 either did not provide physician confirmation of diagnosis, or did not meet the eligibility criteria based on the information provided by their physician on the diagnosis confirmation form, and 32 participants (10%) were interviewed.

Descriptive statistics were used to analyze the sociodemographic and health information. The frequency at which concepts emerged during the interviews, the proportion of participants expressing each concept, and the saliency of concepts elicited in both groups were calculated. Responses to hypothetical scenarios were tabulated and participant quotes were analyzed to extract key messages from the data and to summarize participants’ treatment priorities and factors driving endpoint preferences and their meaningfulness.

The transcripts from anonymized participant interviews were reviewed and coded using ATLAS.ti (version 8.0) software. Qualitative data from the interviews were analyzed using both deductive and inductive coding techniques. Deductive coding was used for those themes and concepts identified *a priori*, i.e. in the literature review and clinician interviews. Inductive coding was also employed, specifically for those concepts and themes that were newly derived from the patient interviews. This combined approach ensured that the coding structure necessary for content analysis of concepts used in the saturation and saliency analyses captured all concepts discussed by patients, while also providing researchers an opportunity to thematically analyze new concepts and ideas as they emerged spontaneously from the patient data [35, 36]. Two researchers independently coded each transcript and discussed changes to the codebook and coding rules until an inter-coder agreement of at least 0.7 was reached, which occurred after the eighth transcript. The frequency of concepts was cross-checked with live capture sheets that were filled in during the interviews by the interviewers.

## Results

### Patient population

Demographic data for the two patient groups comprising 32 participants with advanced or metastatic disease (31 women, 1 man; 17 participants with TNBC and 15 with HR+/HER2− breast cancer) are shown in Table 1. Participants were of diverse origin (13% were Black or African American and 13% were Asian or mixed ethnic groups) and had different experiences with the disease and treatments: 41% of participants in the TNBC group and 53% of those in the HR+/HER2− breast cancer group were first diagnosed with early-stage breast cancer and progressed later to metastatic disease; 35% of participants in the TNBC group and 53% of those in the HR+/HER2− breast cancer group had received second- or third-line treatment; 82% of participants with TNBC had received chemotherapy; 67% of participants with HR+/HER2− breast cancer were treated with just endocrine therapy, while 33% had received both chemotherapy and endocrine therapy.

**Table 1:** Demographic and clinical characteristics of study participants.

	Study participants	
Characteristic	TNBC (n = 17)	HR+/HER2− breast cancer (n = 15)
Age, years, n (%)		
≤ 39	1 (6)	5 (33)
40–49	4 (24)	4 (27)
50–64	9 (53)	6 (40)
65+	3 (18)	0 (0)
Age, years, median (range)	53 (35−72)	48 (32–63)
Race, n (%)		
White	11 (65)	13 (87)
Black or African American	3 (18)	1 (7)
Asian or Pacific Islander	0 (0)	1 (7)
Mixed/multiple ethnic groups	3 (18)	0 (0)
Education, n (%)		
High school diploma	3 (18)	0 (0)
Vocational training	1 (6)	0 (0)
Some college	6 (35)	4 (27)
Associate degree	1 (6)	0 (0)
Bachelor’s degree	4 (24)	7 (47)
Graduate degree	2 (12)	4 (27)
Initial disease stage at diagnosis, n (%)		
I	0 (0)	2 (13)
IIA	5 (29)	2 (13)
IIB	2 (12)	4 (27)
IIIB	1 (6)	0 (0)
IIIC	2 (12)	1 (7)
IV	6 (35)	6 (40)
Unknown (pre-stage IV)	1 (6)	0 (0)
Current disease stage, n (%)		
IIIB	1 (6)	0 (0)
IIIC	2 (12)	0 (0)
IV	14 (82)	15 (100)
Time since diagnosis of advanced/metastatic disease, n (%)		
0−6 months	5 (29)	1 (7)
7−12 months	4 (24)	3 (20)
13−24 months	4 (24)	2 (13)
25−36 months	2 (12)	1 (7)
> 36 months	2 (12)	6 (40)
Unknown	0 (0)	2 (13)
Lines of treatment received for advanced/metastatic disease, n (%)^a^		
1	11 (65)	7 (47)
2	3 (18)	4 (27)
3	3 (18)	4 (27)
Treatment(s) received for advanced/metastatic disease, n (%)		
Chemotherapy only^b^	14 (82)	0 (0)
Endocrine-based therapy only^b^	0 (0)	10 (67)
Both chemotherapy and endocrine-based therapy^b^	0 (0)	5 (33)
Targeted therapy/immunotherapy only	1 (6)	0 (0)
Unknown	2 (12)	0 (0)

^a^Including current line.

^b^With or without targeted therapy/immunotherapy.

HR+/HER2− BC, hormone receptor-positive/human epidermal growth factor receptor 2-negative breast cancer; TNBC, triple-negative breast cancer;

### Signs, symptoms, and impacts

Interviews were conducted in six waves in the TNBC group and in five waves in the HR+/HER2− breast cancer group. Most of the symptom concepts (78 out of 92 in patients with TNBC and 65 out of 75 in patients with HR+/HER2− breast cancer) were formulated in the first two waves of interviews involving six participants from each group (S1 Table). After wave 5, saturation of concepts was reached in the TNBC group. In the HR+/HER2− breast cancer group, three new symptom concepts (food sensitivity, balance issues, and blood pressure fluctuations) emerged and, therefore, saturation was not fully reached; however, these new symptom concepts were deemed uncommon and no further interviews were completed.

Of the 12 most salient symptoms identified across both patient groups (Table 2), fatigue/tiredness and pain were the most bothersome symptoms for 97% and 72% of all participants, respectively (average bother rating 8), followed by difficulty concentrating, hot flashes (especially in HR+/HER2− breast cancer group), memory loss, nausea, and sleep problems (average bother rating 7 for all). Pain, including bone pain and/or muscle and joint pain, was reported by 59% of participants with TNBC and 87% of those with HR+/HER2− breast cancer.

**Table 2:** Salient symptoms^a^ and impacts reported by patients with TNBC and HR+/HER2− breast cancer.

n (%)	TNBC	HR+/HER2− breast cancer	Total	Average bother rating
**Symptoms**				
Fatigue/tiredness/poor energy level	16 (94)	15 (100)	31 (97)	8
Pain (unspecified location)	10 (59)	13 (87)	23 (72)	8
Difficulty concentrating	15 (88)	13 (87)	28 (88)	7
Hot flashes	8 (47)	13 (87)	21 (66)	7
Memory loss	9 (53)	12 (80)	21 (66)	7
Nausea/feeling queasy	10 (59)	10 (67)	20 (63)	7
Sleep problems	11 (65)	9 (60)	20 (63)	7
Alopecia/hair loss	15 (88)	12 (80)	27 (84)	6
Diarrhea	9 (53)	11 (73)	20 (63)	6
Changes in nails/nails weaken	13 (76)	11 (73)	24 (75)	5
Loss of appetite	12 (71)	11 (73)	23 (72)	5
Change in taste	11 (75)	11 (73)	22 (69)	5
**Impacts**				
Worry about the future	16 (94)	15 (100)	31 (97)	8
Anxiety	14 (82)	12 (80)	26 (81)	8
Financial impact	12 (71)	13 (87)	25 (78)	8
Work productivity/ employment	13 (76)	12 (80)	25 (78)	8
Sadness/depression/crying	11 (65)	12 (80)	23 (72)	8
Physical functioning	10 (59)	12 (80)	22 (69)	7
Relationships	10 (59)	11 (73)	21 (66)	7
Social functioning	13 (76)	11 (73)	24 (75)	6
Body image problems	10 (59)	11 (73)	21 (66)	6

^a^Symptoms reported by > 60% of participants and with a rating of ≥ 5 on the disturbance scale (0 = not bothersome; 10 = extremely bothersome). HR+/HER2− BC, hormone receptor-positive/human epidermal growth factor receptor 2-negative breast cancer; TNBC, triple-negative breast cancer.

Several symptoms were mentioned more frequently by participants with HR+/HER2– breast cancer than by participants with TNBC. Vaginal dryness was mentioned by 87% of participants with HR+/HER2– breast cancer (average bother rating 6.5) and 35% of those with TNBC (average bother rating 6). Mouth sores/ulcers were mentioned by 80% of participants with HR+/HER2– breast cancer (average bother rating 5) versus 18% of those with TNBC (average bother rating 9). Headaches/head pain was mentioned by 67% of participants with HR+/HER2– breast cancer (average bother rating 5.8) versus 24% of those with TNBC (average bother rating 5).

Participants reported more than 40 different impacts affecting everyday life (S2 Table). The most salient impacts had comparable frequencies in both groups; they included worrying about the future, anxiety, financial impacts, work productivity and employment, social and physical functioning, relationships, and body image issues; 80% of patients with HR+/HER2− breast cancer and 65% of those with TNBC reported feeling sad or depressed (Table 2).

### Treatment priorities: Patient-tailored treatments

Similar themes emerged in both patient groups in discussions of patient-tailored treatments and priorities; exemplary quotes are presented in Table 3. Most participants across both groups first and foremost wanted a treatment that would cure their disease or put them in remission. The next treatment priority was minimal side effects (66%), followed by maintained/improved HRQoL (38%). Furthermore, 22% of participants considered the mode of administration important, preferring pills over injections, and 19% indicated a preference for a mechanism of action targeting specifically cancer cells over a treatment affecting all cells.

**Table 3:** Exemplary quotes.

Concepts	Exemplary quotes
Patient-tailored treatment: patients considering the relevance of the available information and trade-offs between efficacy and side effects	*“What is the progression-free survival rate*, *what are the major side effects*, *what is the toxicity*, *how long has it been in market*, *what are the results of the trials*, *and what is the overall survival rate*?*”* *“As much as a drug can prolong life … life’s not worth living if you absolutely can’t do anything*, *if you’re stuck vomiting 24 hours a day*, *there’s not a reason for life-prolonging medication at that time*.*”*
Treatment priorities: patients expressing their preference for maintaining the HRQoL and consistency of treatment outcomes (Fig 1)	*“What’s important to me is my quality of life*, *my activity level*, *being able to go to the gym*, *being able to be out in public*.*”* *“But if both scenarios*, *if both of the drugs are working as they’re supposed to in both patients*, *I would still rather have it where it didn’t affect how I felt on a day-to-day basis*.*”* *“Because at least with patient [scenario] B [Fig 1B, no change from baseline] it’s not unpredictable. This patient kind of knows that there is a pretty steady treatment plan.”*
Treatment priorities: stability as the preferred treatment outcome (Fig 1)	*“I guess B again [Fig 1B, no change from baseline]. I just don’t like the ups and downs.”* *“Because I feel like if you have no changes [in HRQoL]*, *but the drugs are actually working*, *you can go about your life as normal […] Having none of that change and just feeling your normal self would be the best scenario*.*”*
Treatment priorities: patients’ concerns about treatment efficiency in the absence of change in how they are feeling (Fig 1)	*“Even though there’s ups and downs it’s still trending up and there’s more okay than bad. Whereas patient [scenario] B [Fig 1B, no change from baseline] I might kind of question: well, what was it doing if you feel the same from beginning to end? It’s good that they’re not feeling worse, but is it effective?”*
Treatment priorities: willingness to accept some toxicity if it is followed by improvement, but not deterioration at the end of the trial (Fig 1)	*“If you told me it was going to be bad upfront but then maybe [I was] feeling better*, *I would feel positive about what I have to look forward to*.*”* *“I would much rather feel worse in the beginning of a treatment*, *knowing that*, *okay*, *I’m feeling worse probably because my body is acclimating to it*. *Then once my body acclimates to it*, *I kind of get back to where I was feeling at the beginning and know that*, *alright*, *I can ride this out now*.*”* *“Because they feel like crap at the end*. *That whole ending thing*. *I can’t get with the feeling so great and then you tank and then you’re questioning ‘Why in the hell did I ever do that’*? *If I’ve gotta go with the status quo*, *I’m going to maintain my same quality of life because I know what to expect*. *I know my limitations*.*”*
Endpoint preference: average score of physical function over time (Fig 2A)	*“I think people understand average more than anything*.*”* *“It just more easily shows me that over the duration–so there’s already more information there–that they were so similar in the beginning*, *but then the treatment in orange goes above the blue line*. *Just more visually accepting to me personally*.*”* *“I would actually prefer the other graph with the lines on it [Fig 2A], only because it does include the timeline of the trial or the treatment plan. You kind of get more of the bigger picture than just…. Was this bar graph made in the first month, or was it made after the first year? I don’t know the timeline of when these results were taken.”*
Endpoint preference: group-level trial outcome data about physical function (Fig 2B)	*“This second one [Fig 2B] is a lot easier to grasp and you see the benefits a lot more clearly … I would say, combined with an explanation that in both types of treatment, you should expect or most people experience first worsening and then improvement, particularly in the second year.”* *“It’s nice to see the end result too … I like knowing by the end of this particular treatment how am I going to be doing*. *To me*, *that would be important information*.*”* *“I guess, for me, I would like to see both of them [Fig 2A and 2B] because it does give a different perspective on seeing the progress month by month or seeing by the end. “*
“Time to worsening” versus “maintaining quality of life” time wording	*“‘Time to worsening*,*’ I wish there was a better phrase*. *I mean*, *I know what they’re trying to say*, *but it’s like you had an improvement of quality … like you felt better for 23 months*. *[…] You’re maintaining or improving your quality of life for 23 months instead of 16 months*, *you know*? *[… ] And it’s also a recognition to patients that it’s not just quantity of life*, *it’s quality of life*.*”*
TNBC and HR+/HER2− breast cancer differences in perceptions about meaningful change in time to deterioration (Fig 3)	*“She [the oncologist] had said at least 10 years*, *which I don’t know that that’s accurate*. *Nobody knows*, *but 7 months in a 10-year time frame is not a big deal for a small thing like doing strenuous activities… I would say maybe a year difference would be helpful*.*”* **(Participant with HR+/HER2− breast cancer)** *“I’d probably like 3 months*. *I think 7 months is really … that would be great*. *But I’m thinking that even as little as 3 months would be something I would be willing to go for*.*”* **(Participant with TNBC)**

When describing key information that would facilitate their decision-making regarding a new treatment, 81% of participants in both groups centered their responses on toxicity and the lasting effects. The side effects that participants most wanted to avoid were nausea (38%), fatigue (34%), and hair loss (28%). Thirty-four percent of participants would also ask about the known survival rate and availability of prior trial results; 31% wanted to know the expected outcomes at the end of treatment.

### Treatment priorities: Best treatment outcomes

When presented with different variants of HRQoL trial responses, participants in both groups chose scenario B (no change in HRQoL, Fig 1B) in 74 of the 96 presentations (presented three times to each participant), regardless of which other scenario was given for comparison. Nevertheless, even in the context of preferred stability and consistency of the HRQoL, treatment efficacy remained a high priority. No obvious worsening in HRQoL over time was interpreted by participants as a sign of no underlying disease progression; however, for some of them, it raised a concern around a lack of treatment efficacy (Table 3).

The other favorable treatment responses for both groups were presented in scenario C, which involved initial deterioration in HRQoL followed by improvement and subsequent return to baseline, and in scenario E, which had a short period of deterioration followed by constant HRQoL at a baseline level (Fig 1C and 1E). Almost two-thirds of participants (65%) favored these two scenarios because they appreciated return to baseline HRQoL and viewed the initial worsening as an expected adjustment to the new drug, which resonated with their previous experiences with other treatments (Table 3).

Only 13% of participants favored scenarios A and D that resulted in a worsening of HRQoL at the end of treatment (Fig 1A and 1D). These participants described some degree of benefit from a period of improved HRQoL, allowing them to perform more daily activities and tasks than they normally would have done, especially if it was for a significant period of time (e.g. longer than 6 months, as was pointed out by one of the participants with HR+/HER2− breast cancer). Only three participants from the TNBC group and two from the HR+/HER2− breast cancer group preferred scenario A because they liked the prolonged period of increased HRQoL that preceded the worsening; however, for most participants, the increase was not worth the ensuing deterioration (Table 3).

### Endpoint preference exercise

In the endpoint preference exercise, participants found a plot of the mean score of physical function over time (Fig 2A) generally easy to interpret. Participants also appreciated that this scenario allowed them to compare treatments over time, from baseline to the end of the trial. The endpoint presentation as the proportion of participants who improved, had no change, or experienced decline of their physical function over the course of treatment (Fig 2B) was also viewed favorably by both groups of participants (Table 3), who found that the information grouped in this way made it relatively easy to compare the two treatments and to set expectations. However, they highlighted as a limitation that this endpoint did not show a change in physical function status over time, in contrast to changes in mean physical score shown in Fig 2A. Some participants said that both changes over time and physical function at the end of the trial were important (Table 3).

The median “time to worsening” endpoint that showed time until the level of physical function decreased for 50% of patients in the course of the trial (Fig 2C) was also viewed by many participants as useful because it showed how long they would feel “normal” and could help to plan for their future. However, some participants found it difficult to grasp that this representation showed time until worsening for 50% of the trial patients (median) and not for the entire sample. The exact meaning of “time to worsening” was also not immediately apparent to some participants; some interpreted it as the duration of worsening of the symptoms. Participants suggested that changing the wording to “maintaining quality of life time” may be better understood.

**Fig 3:**
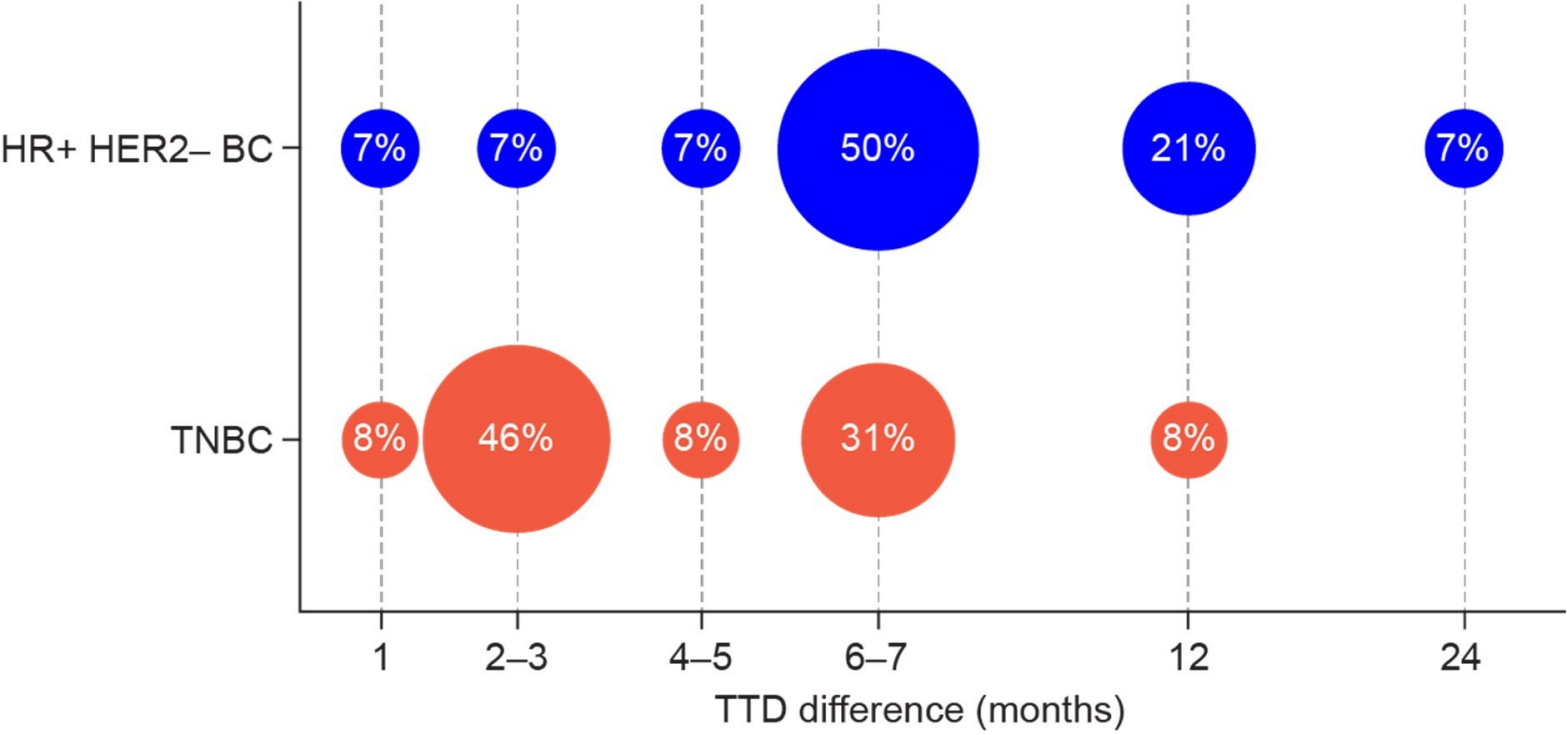
Minimum meaningful TTD difference in physical functioning by breast cancer sub-type.

The minimum TTD in physical function that was considered significant by participants with TNBC and HR+/HER2− breast cancer is shown in Fig 3. While 46% of participants with TNBC indicated that a difference of 2–3 months of preserved quality of life time was significant for them, more than 50% of participants with HR+/HER2− breast cancer viewed a relatively longer period, starting at 6–7 months, as meaningful (Table 3).

Overall, when evaluating endpoints for their meaningfulness for treatment decision-making on a 10-point scale, presentation of trial outcome data in Fig 2B (percentage of patients with different outcomes) received the highest average rating of 7.8, while change in mean score for physical function from baseline (Fig 2A) had an average rating of 6.7, and “time to worsening” had an average rating of 6.6. HR+/HER2− BC, hormone receptor-positive/human epidermal growth factor receptor 2-negative breast cancer; TNBC, triple-negative breast cancer; TTD, time to worsening (corresponding to time to deterioration).

## Discussion

Engaging patients in a direct conversation about their personal experiences facilitates the development of methods of capturing information that are needed to inform drug development, clinical trial design, and regulatory decision-making. It also aids understanding of the potential acceptability of trade-offs between the benefits and risks of treatment, and helps patients to set realistic expectations. The need for guidance on appropriate endpoints and incorporation of patient-reported outcomes in clinical trials is underscored by the fact that several recent randomized controlled trials in advanced breast cancer have yielded statistically significant improvements in the primary endpoint with experimental therapy but have not lead to regulatory approval or practice change, primarily due to toxicity burden and unfavorable risk/benefit profiles [37, 38].

Whereas a conceptual model of the signs, symptoms, and impacts of HR+/HER2− breast cancer has been previously published [17], corresponding information for patients with TNBC is limited, and it was informative to evaluate and to compare the perceptions of these two clinically distinct groups of patients with different treatment experiences and prognoses. The concept elicitation portion of the interviews revealed that participants with TNBC and those with HR+/HER2− breast cancer have similar symptoms and impacts of disease on their lives. In line with previously published data, fatigue/tiredness and pain were identified as the most bothersome symptoms [18, 19]. One notable difference was that participants with HR+/HER2− breast cancer mostly experienced pain in the joints and attributed it to the effects of treatment, whereas participants with TNBC mainly suffered from breast pain and considered it to be an effect of the disease. Also, more participants with HR+/HER2− breast cancer than those with TNBC reported feeling sad or depressed; high rates of self-reported symptoms of depression among patients with HR+ breast cancer have been reported previously [17, 39]. Other reported differences between the two patient populations (i.e. more participants with HR+/HER2– breast cancer reported vaginal dryness, mouth sores/ulcers, and headaches than participants with TNBC) speak to the heterogeneity of symptom presentation and classes of treatment deployed between these populations.

While some studies have shown that, even among educated, heavily pretreated patients, many commonly used clinical research terms are poorly understood [7], our results demonstrate that discussion of hypothetical scenarios allows patients to adequately interpret and speak about graphical representations of different therapeutic effects and HRQoL-related endpoints. As expected, both groups of participants preferred to receive treatment that is effective and long-lasting in preserving HRQoL. The paramount importance of maintaining an acceptable level of HRQoL across the remainder of a patient’s life in advanced stages of cancer is known from previous studies [1, 25].

Although experiencing fewer less serious and non-permanent side effects, and avoiding specific side effects, such as nausea and fatigue, were the priorities, most participants were willing to accept a moderate level of toxicity as long as there was consistency in their level of well-being or an eventual return to baseline after a transient period of deterioration. The predictability of HRQoL during treatment was also a recurring theme: fluctuations in treatment experience were viewed negatively; patients valued treatments that prolonged periods of normalcy; and most of them did not favor treatments that were associated with a temporary improvement if it was followed by deterioration.

When comparing different endpoints, participants wanted to know both the expected proportion of cases that would experience improvement, deterioration, or stability of the symptoms during and at the end of treatment, and the mean changes in physical function score during the trial; at the same time, although “time to worsening” (corresponding to TTD) information was deemed useful, this terminology was less favorable. The perception of “time to worsening” could have been affected by the “negative” wording; the patients suggested phrasing it more positively and focusing on “preserved HRQoL time”. Positively phrased parameters are known to be associated with increased perceived importance by patients [7]; reframing “time to worsening” to focus instead on “maintaining quality of life time” may be a better way of presenting this information.

When considering TTD, the notion of a “meaningful” period was longer for patients with HR+/HER2− breast cancer than for those with TNBC, which was probably influenced by the differences in life expectancy and treatment toxicity profiles. This distinction in perception of meaningful TTD by target populations should warrant a more tailored approach to communicating treatment information to patients and should be considered during drug development.

There are some limitations to this study. Concept saturation was not achieved in patients with HR+/HER2− breast cancer. Although the study’s sample size was estimated to reach the primary objective through concept saturation, this study is exploratory and did not recruit a large enough sample to conduct or achieve saturation of the secondary objectives. For the secondary objectives, these data should be considered informative and hypothesis generating. The hypothetical scenarios were designed to elicit patient views on selecting preferred scenarios based on their personal preferences, with a focus on identifying themes based on patients’ explanations of the reasons behind their choices. However, by necessity, the scenarios were simplifications of treatment outcomes and did not represent all factors that patients and physicians need to evaluate when making treatment decisions. Furthermore, the relatively small cohort size might have resulted in selection bias and lack of representativeness; however, consistency of the main results in two clinically different participant groups indicates that they likely can be generalized to the broader advanced breast cancer population. Non-participants may have differed from participants in terms of education, age, race, and time since diagnosis, which in turn could have affected the total mention of symptoms and the patients’ abilities to interpret scenarios and graphs. Clinical trials targeting patients with cancer should capture disease- and treatment-related symptoms and impacts that are most important to patients, to provide a complementary view to traditional efficacy and safety outcomes. Finally, understanding what patients deem as meaningful outcomes of their treatment regimens is important and should be considered by regulatory bodies.

## Conclusions

Patients with advanced-stage TNBC and those with HR+/HER2− breast cancer clearly identified similar priorities, indicating efficacy, tolerability, and stability and predictability in their HRQoL as critical factors when considering a new treatment. The results of this study may help ensure clinical trials include endpoints that capture information identified as meaningful to patients.

## Supporting information

S1 Table Signs and symptoms referred to by participants with TNBC and HR+/HER2− breast cancer. (DOCX)

S2 Table Impacts reported by participants with TNBC and HR+/HER2− breast cancer. (DOCX)

S1 Checklist COREQ checklist. (DOCX)
