# Supplementary material for: Using qualitative interviews to identify patient-reported clinical trial endpoints and analyses that are the most meaningful to patients with advanced breast cancer
Source: PLoS One. 2023 Jan 17;18(1):e0280259. doi: 10.1371/journal.pone.0280259 (PMC9844842; doi:10.1371/journal.pone.0280259)
Supplement: S1 Table — (DOCX) [file pone.0280259.s001.docx]

**Supplement Table 1** Signs and symptoms referred to by patients with TNBC and HR+/HER2– breast cancer

| **Symptoms** | **Patients with  TNBC** | **Patients with HR+/HER2– breast cancer** |
| --- | --- | --- |
| Acid reflux/heartburn | X | X |
| Alopecia/hair loss | X | X |
| Anemic/low blood cell count/looking pale | X | X |
| Appetite gain |  | X |
| Balance issues | X | X |
| Bleeding | X |  |
| Blood pressure fluctuations | X | X |
| Blood sugar fluctuations | X |  |
| Blurry vision | X | X |
| Bone pain | X | X |
| Bruising | X | X |
| Burning in extremities | X | X |
| Cachexia (muscle loss) | X |  |
| Capsular contracture (breast implant) | X |  |
| Change in taste | X | X |
| Changes in nails/nails weaken | X | X |
| Clot | X |  |
| Cold flashes/chills | X | X |
| Constipation | X | X |
| Cough | X | X |
| Decrease in vision | X | X |
| Decreased ability to perceive sensations (e.g., pain, heat) | X | X |
| Dehydration |  | X |
| Diarrhea | X | X |
| Difficulty concentrating/learning | X | X |
| Difficulty swallowing | X | X |
| Digestion issues/feeling full | X |  |
| Discharge from the nipple | X |  |
| Discomfort | X | X |
| Discomfort, ‘Feeling of something being attached to my chest wall’ | X |  |
| Dizziness/light-headedness | X | X |
| Drowsiness |  | X |
| Dry eyes | X | X |
| Dry mouth | X | X |
| Dry skin | X | X |
| Dyspareunia/painful vaginal intercourse |  | X |
| Elevated resting heart rate | X |  |
| Esophagitis/burning throat |  | X |
| Fatigue/tiredness and feeling weak | X | X |
| Feeling bloated/flatulence (farting) | X | X |
| Fever | X | X |
| Flu-like symptoms | X | X |
| Food sensitivity | X | X |
| Frequent urination | X | X |
| Frozen shoulder syndrome | X |  |
| Gravelly voice/hoarseness | X |  |
| Grogginess | X |  |
| Hand-foot syndrome | X | X |
| Hand tremors/shaking | X |  |
| Headaches | X | X |
| Hot flashes | X | X |
| Infection | X | X |
| Inverted nipple |  | X |
| Irregular periods | X | X |
| Irritated eyes/redness of eyes | X | X |
| Itching | X | X |
| Joint pain | X | X |
| Loss of appetite | X | X |
| Lump in collarbone | X |  |
| Lump/mass of the breast or under the arm | X | X |
| Memory loss | X | X |
| Menopausal symptoms | X | X |
| Mouth sores/ulcers | X | X |
| Muscle pain | X | X |
| Muscle spasms | X |  |
| Muscle weakness | X |  |
| Nausea/feeling queasy | X | X |
| Neuropathy (burning/tingling/numbness) | X | X |
| Night sweats | X | X |
| Pain elsewhere in the body (aches, back pain, bone pain, fractures, leg, chest, shoulder, pain down the side of body) | X | X |
| Pain from hair loss | X |  |
| Pain in the breast | X | X |
| Polyps on eyelids | X |  |
| Ringing in the ears/tinnitus | X |  |
| Sensitive/tender breast | X | X |
| Shortness of breath/difficulty breathing | X | X |
| Skin burns | X |  |
| Skin changes on/in affected breast | X | X |
| Skin color change | X | X |
| Skin nodules | X |  |
| Skin rash | X | X |
| Skin spots | X | X |
| Sleep problems | X | X |
| Stiffness | X | X |
| Stomach discomfort/stomach pain |  | X |
| Swelling of arms/legs/hands/feet/face | X | X |
| Swollen breast/change in size or shape of breast | X | X |
| Teeth/gums problems | X |  |
| Thickening of the breast or under the arm | X | X |
| Tingling/numbness in extremities | X | X |
| Trouble eating | X |  |
| Vaginal discharge | X |  |
| Vaginal dryness | X | X |
| Vertigo |  | X |
| Visual flashes | X |  |
| Visual floaters | X |  |
| Vomiting | X | X |
| Weak bones | X | X |
| Weight gain | X | X |
| Weight loss | X | X |
| Wet eyes |  | X |

X signifies that a patient referred to the particular sign or symptom
