# Supplementary material for: Using qualitative interviews to identify patient-reported clinical trial endpoints and analyses that are the most meaningful to patients with advanced breast cancer
Source: PLoS One. 2023 Jan 17;18(1):e0280259. doi: 10.1371/journal.pone.0280259 (PMC9844842; doi:10.1371/journal.pone.0280259)
Supplement: S2 Table — (DOCX) [file pone.0280259.s002.docx]

**Supplement Table 2** Impacts reported by patients with TNBC and HR+/HER2– breast cancer

| **Impacts** | **Patients with TNBC** | **Patients with HR+/HER2– breast cancer** |
| --- | --- | --- |
| Anxiety | X | X |
| Avoiding large crowds | X |  |
| Changed alcohol drinking habits | X | X |
| Changed diet | X | X |
| Cognitive problems | X | X |
| Dependency on others | X | X |
| Depression/sadness/crying | X | X |
| Developing positive attitude/reduced worriedness | X | X |
| Difficulty doing daily activities | X | X |
| Difficulty doing household chores and groceries (cooking, cleaning, laundry, folding clothes, etc.) | X | X |
| Difficulty dressing, undressing, showering | X | X |
| Difficulty driving/avoiding driving | X | X |
| Difficulty sitting for too long |  | X |
| Feeling of guilt | X |  |
| Feeling out of place, having an old person disease | X |  |
| Financial impact | X | X |
| Having to stay in bed | X | X |
| Impact on physical function | X | X |
| Impact on relationships/marriage – negative | X | X |
| Impact on relationships/marriage – positive | X | X |
| Impact on role function | X | X |
| Impact on sexual activities | X | X |
| Impact on social function | X | X |
| Impact on work productivity and employment | X | X |
| Inconvenience of current treatment | X | X |
| Lack of focus/attention/difficulty reading | X | X |
| Limited range of movement | X | X |
| Mood swings/irritability | X | X |
| Painful intercourse | X | X |
| Reduced ability to help/support family | X |  |
| Reduced sex drive | X | X |
| Reduced strength | X | X |
| Self-consciousness/low self-esteem | X | X |
| Sleep disturbance | X | X |
| Stress/nervousness | X | X |
| Trouble climbing up stairs | X | X |
| Trouble doing exercise/sports | X | X |
| Trouble moving | X | X |
| Trouble standing | X | X |
| Trouble walking | X | X |
| Unable to attend doctor appointments |  | X |
| Unable to do fun things / leisure activities / outdoor activities | X | X |
| Unhappiness with bodily appearance | X | X |
| Worry about the future | X | X |

X signifies the presence of an impact
