# Supplementary material for: Using qualitative interviews to identify patient-reported clinical trial endpoints and analyses that are the most meaningful to patients with advanced breast cancer
Source: PLoS One. 2023 Jan 17;18(1):e0280259. doi: 10.1371/journal.pone.0280259 (PMC9844842; doi:10.1371/journal.pone.0280259)
Supplement: S1 Checklist — (DOCX) [file pone.0280259.s003.docx]

COREQ checklist

| **Topic** | **Item No.** | | **Guide Question/Description** | | **Reported on Page** |
| --- | --- | --- | --- | --- | --- |
| **Domain 1: Research Team and Reflexivity** | | | | | |
| *Personal Characteristics* | | | | | |
| Interviewer/Facilitator | | **1** | | Which author/s conducted the interview or focus group? | 9 |
| Credentials | | **2** | | What were the researcher's credentials? E.g., PhD, MD | 9 |
| Occupation | | **3** | | What was their occupation at the time of the study? | 9 |
| Gender | | **4** | | Was the researcher male or female? | 9 |
| Experience and Training | | **5** | | What experience or training did the researcher have? | 9 |
| *Relationship with Participants* | | | | | |
| Relationship Established | | **6** | | Was a relationship established prior to study commencement? | 9 |
| Participant Knowledge of the Interviewer | | **7** | | What did the participants know about the researcher? e.g., personal goals, reasons for doing the research | 9 |
| Interviewer Characteristics | | **8** | | What characteristics were reported about the interviewer/facilitator? e.g., Bias, assumptions, reasons and interests in the research topic | 9 |
| **Domain 2: study design** | | | | | |
| *Theoretical Framework* | | | | | |
| Methodological Orientation and Theory | | **9** | | What methodological orientation was stated to underpin the study? e.g. grounded theory, discourse analysis, ethnography, phenomenology, content analysis | 9 |
| *Participant Selection* | | | | | |
| Sampling | | **10** | | How were participants selected? e.g. purposive, convenience, consecutive, snowball | 9 |
| Method of Approach | | **11** | | How were participants approached? e.g. face-to-face, telephone, mail, email | 9 |
| Sample Size | | **12** | | How many participants were in the study? | 13 |
| Non Participation | | **13** | | How many people refused to participate or dropped out? Reasons? | 13 |
| *Setting* | | | | | |
| Setting of Data Collection | | **14** | | Where was the data collected? e.g. home, clinic, workplace | 9 |
| Presence of Non-Participants | | **15** | | Was anyone else present besides the participants and researchers? | 9 |
| Description of Sample | | **16** | | What are the important characteristics of the sample? e.g. demographic data, date | 9 |
| *Data collection* | | | | | |
| Interview Guide | | **17** | | Were questions, prompts, guides provided by the authors? Was it pilot tested? | 9 |
| Repeat Interviews | | **18** | | Were repeat interviews carried out? If yes, how many? | 9 |
| Audio/Visual Recording | | **19** | | Did the research use audio or visual recording to collect the data? | 9 |
| Field Notes | | **20** | | Were field notes made during and/or after the interview or focus group? | 9 |
| Duration | | **21** | | What was the duration of the interviews or focus group? | 9 |
| Data Saturation | | **22** | | Was data saturation discussed? | 9 |
| Transcripts Returned | | **23** | | Were transcripts returned to participants for comment and/or correction? | 9 |
| **Domain 3: Analysis and Findings** | | | | | |
| *Data Analysis* | | | | | |
| Number of Data Coders | | **24** | | How many data coders coded the data? | 13 |
| Description of the Coding Tree | | **25** | | Did authors provide a description of the coding tree? | 13 |
| Derivation of Themes | | **26** | | Were themes identified in advance or derived from the data? | 13 |
| Software | | **27** | | What software, if applicable, was used to manage the data? | 13 |
| Participant Checking | | **28** | | Did participants provide feedback on the findings? | 13 |
| *Reporting* | | | | | |
| Quotations Presented | | **29** | | Were participant quotations presented to illustrate the themes / findings? Was each quotation identified? e.g. participant number | 21-24 |
| Data and Findings Consistent | | **30** | | Was there consistency between the data presented and the findings? | 14-26 |
| Clarity of Major Themes | | **31** | | Were major themes clearly presented in the findings? | 14-26 |
| Clarity of Minor Themes | | **32** | | Is there a description of diverse cases or discussion of minor themes? | 20 |
